# Characterization of a population of transgender individuals and their perceived negative mental health and life experiences

**DOI:** 10.1192/j.eurpsy.2023.1344

**Published:** 2023-07-19

**Authors:** M. F. Santos, J. Mota, Z. Figueiredo

**Affiliations:** Department of Psychiatry, Hospital de Magalhães Lemos, Porto, Portugal

## Abstract

**Introduction:**

Gender dysphoria (GD) is characterized by a marked incongruence between one’s experienced/expressed gender and gender assigned at birth, associated with clinically significant distress or impairment in important areas of functioning. Persons with GD are a population with specific healthcare needs. In our hospital, psychiatric assessment of these individuals started in 2008.

**Objectives:**

Characterization of a population of transgender individuals and their perceived negative mental health and life experiences.

**Methods:**

Since the beginning of evaluations of transgender individuals in our hospital, from 2008 to 2022, they were asked to freely elaborate their “life story”: a report of the most relevant events in their lives, related to their condition. We retrospectively analyzed the content of the reports that were sent and associated with the clinical files.

**Results:**

We collected the data of 104 individuals. The gender attributed at birth consisted of 74 (71.2%) females and 30 (28.8%) males. As for the gender identity, the sample consisted of 73 (70.2%) males, 28 (26.9%) females, 2 (1.9%) nonbinary and 1 (1%) person didn’t identify with any of the existent denominations. The medium age in October of 2022 was 27.4 (minimum 18, maximum 60, SD 7.3). The age at first evaluation at consult was 23.6 (minimum 15, maximum 56, SD 7.2). 99 (95.2%) individuals mentioned symptoms of gender non-conformity beginning in childhood, and of those who mentioned their adolescence (n=43, 41.3%), all expressed feelings of anguish relating to their changing bodies. The medium age of recognition of their condition was 17.2 (minimum 11, maximum 30, SD 4.3). Of those who recall their first contact with health practitioners regarding their symptoms (n=31, 29.8%), 32.3% admitted they didn’t feel they were helped. Of those who mentioned early relationships with family and carers (n=65, 62,5%), 35.4% reveal dysfunctional relationships and 79% mention gender expectations from their families. Similarly, 42 (40.4%) individuals reveal experiences of victimization and bullying because of their gender nonconformity. 53 (60.2%) described symptoms of a likely comorbid psychiatric illness throughout their life, particularly depressive symptoms, anxious symptoms, suicide attempts and non-suicidal self-injury.

**Conclusions:**

GD has gained more attention in the recent years, and the scientific community has now developed a more accurate set of criteria for the recognition of this condition. The findings in our study are in accordance with these criteria. Unfortunately, much of the suffering this condition entails is also associated with distress related to stigma and societal gender expectations, and that was evident in this investigation.

**Disclosure of Interest:**

None Declared

